# Induction Motor Stator Winding Inter-Tern Short Circuit Fault Detection Based on Start-Up Current Envelope Energy

**DOI:** 10.3390/s23208581

**Published:** 2023-10-19

**Authors:** Liting Chen, Jianhao Shen, Gang Xu, Cheng Chi, Qiaohui Feng, Yang Zhou, Yuanzhi Deng, Huajie Wen

**Affiliations:** 1 College of Urban Transportation and Logistics, Shenzhen Technology University, Shenzhen 518118, China; 2100411003@email.szu.edu.cn (L.C.); xugang@szu.edu.cn (G.X.); chicheng@sztu.edu.cn (C.C.); 2110412007@email.szu.edu.cn (Q.F.); zhouyang2017@email.szu.edu.cn (Y.Z.); dengyuanzhi2020@email.szu.edu.cn (Y.D.); wenhuajie2019@email.szu.edu.cn (H.W.); 2 The Shenzhen Key Laboratory of Urban Rail Transit, Shenzhen Technology University, Shenzhen 518118, China

**Keywords:** induction motor, fault detection, inter-tern short circuit, envelope, support vector machine

## Abstract

Inter-turn short circuit (ITSC) is a common fault in induction motors. However, it is challenging to detect the early stage of ITSC fault. To address this issue, this paper proposes an ITSC fault detection method for three-phase induction motors based on start-up current envelope energy. This approach uses Akima interpolation to calculate the envelope of the measured start-up current of the induction motor. A Gaussian window weighting is applied to eliminate endpoint effects caused by the initial phase angle, and the enveloping energy is obtained using the energy formula as the fault feature. Finally, by combining this with the support vector machine (SVM) classification learner, fault detection of ITSC in induction motors is achieved. The experimental results show that the average accuracy of this method reaches 96.9%, which can quickly and accurately detect ITSC faults in asynchronous motors and determine the severity of the faults. Furthermore, the average accuracy of SVM in detecting early ITSC faults under no-load conditions is 98.8%, which is higher than other classification learners, including LR, KNN, and NN. This study provides a new idea for induction motor fault detection and can contribute to induction motor maintenance.

## 1. Introduction

Induction motors are widely used in various industrial fields due to their simple structure, excellent performance, and outstanding cost-effectiveness. However, the effect of the working environment can lead to the occurrence of faults. The faults of induction motors are mainly divided into mechanical faults and electrical faults. Electrical faults include inter-turn short circuit (ITSC), open-circuit, ground, over/under-voltage faults, and so on [1]. Among them, the ITSC fault of the stator winding is one of the most common faults, accounting for 30% to 40% of all induction motor faults [2]. The reason is that electrical and thermal stresses cause damage to the turn-to-turn insulation layer, resulting in ITSC faults. The most evident phenomena in the middle and late stages of ITSC faults of the stator winding are asymmetric phase currents and local overheating of the winding. If the fault further intensifies, it will cause motor damage and even catastrophic accidents in the entire system. Therefore, it is necessary to have a technology that can timely detect the ITSC fault and take appropriate measures to ensure the safe operation of the equipment. The former fault detection methods, which were based on user reviews, required monitoring the visual and auditory information gathered from the equipment to assess the motor’s health [3]. However, this approach incurred substantial costs in terms of human labor and time. Currently, the main researched fault detection technologies include motor current signature analysis (MCSA) [4,5,6,7,8], vibration analysis [3,9,10], acoustic signal analysis [11,12,13], and thermal imaging analysis [14,15,16]. Jung et al. [4] proposed an online diagnostic method for current signature analysis based on advanced signal- and data-processing algorithms, which successfully achieved the diagnosis of three types of faults, namely rotor misalignment, stator winding short circuit, and bearing defects, in induction motors. Cusidó et al. [5] proposed a current signature analysis method based on injecting signals of different frequencies to identify faults in induction motors, such as rotor broken bars, bearing damage, and rotor eccentricity, which provides new ideas for fault diagnosis in electric motors. Bouzida et al. [6] proposed a fault diagnosis method based on the discrete wavelet transform, which extracts the health information of electric motors from wide-band signals utilizing wavelet decomposition. Ciszewski et al. [7] proposed a current feature analysis diagnosis method based on higher-order spectral technology, which effectively identified bearing faults in induction motors. Kim et al. [3] proposed a fault diagnosis method based on vibration signals, and utilized various machine learning models to achieve the classification of motor health, rotor faults, and bearing faults. Kafeel et al. [8] proposed a fault detection system based on vibration signal analysis, which utilized empirical mode decomposition for multi-domain feature extraction and achieved satisfactory results through experiments. Pham et al. [10] proposed a deep learning-based bearing fault diagnosis method using acoustic emission signals, which has the advantages of higher accuracy and lower computational complexity compared to previous deep learning-based diagnostic methods. Alvarado-Hernandez et al. [13] proposed a fault monitoring technology based on infrared thermal imaging intelligent sensors, which was validated on an experimental test bench. To sum up, the characteristic of these methods is to extract signals under the stable operating state of the motor. However, in the early stages of ITSC faults in the motor, the fault features extracted from the signal under stable operation are relatively weak, especially under the influence of interference factors such as no-load, unstable power supply voltage, and noise, which can bring great difficulties to the fault detection of the motor.

During the start-up process, the induction motor operates under more critical conditions, where the current reaches two to three times that of the stable operation stage, which helps to amplify the characteristics of early faults [17]. Multiple fault detection methods have emerged in the field of transient analysis based on the start-up process [15,17,18,19,20,21,22,23,24,25,26,27,28,29]. For ITSC fault detection, Singh et al. [15] proposed an ITSC fault detection method using an infrared thermal imager, which detects the transient thermal during the start-up process of an induction motor to assess the severity of the fault present in the motor. Zaparoli et al. [22] proposed a method for detecting ITSC faults based on principal component analysis and monitored the evolution of faults through variance analysis. However, this method uses a threshold to determine the degree of motor failure and does not provide a specific accuracy rate.

Compared with the above studies, our work focuses primarily on detecting early ITSC faults in three-phase induction motors during start-up. We utilize current parameters to study the ITSC fault detection technology in three-phase induction motors and conduct diagnostic evaluations of the fault severity. Based on the envelope and amplitude energy analysis theory and combined with the support vector machine (SVM) classification model, we propose an ITSC fault detection method based on start-up current. To evaluate the effectiveness of our method, we first create a simulation environment where we establish a mathematical model for ITSC faults in three-phase induction motors. We then collect simulation data of starting current signals and apply our proposed method for evaluation. Subsequently, we construct a motor performance testing platform to obtain the actual current signals during the three-phase induction motors’ start-up process and further validate our method’s effectiveness. Our proposed method can detect early ITSC faults during the start-up process of three-phase induction motors, enabling diagnosis before the induction motor enters regular operation. Consequently, it effectively minimizes losses caused by induction motor failures during operation.

The main contributions of this paper are as follows: (1) According to the current variation characteristics during the start-up process of a three-phase induction motor, a detection algorithm for ITSC faults based on the energy of start-up current envelope is proposed. (2) Compare the performance of the proposed method with that of other machine learning classifiers. The former has higher accuracy in detecting ITSC faults and also achieved satisfactory results under no-load conditions.

The rest of the article is organized as follows: Section 2 introduces the mathematical model of ITSC faults in three-phase induction motors. Section 3 illustrates the algorithms for fault detection and provides corresponding explanations for the methods used. To evaluate the detection strategy, relevant simulation studies were conducted in Section 4. Section 5 explains the experiment setup, presents the results, and provides a discussion. A summary of the work is given in Section 6.

## 2. Mathematical Model of Induction Motor ITSC Fault

To evaluate the feasibility of the proposed detection strategies, it is necessary to study the effects of motors under different operating conditions. Therefore, it is crucial to select appropriate induction motor models for simulation. Considering the influence of fault resistance ($R_{f}$) and stator ITSC, Tallam’s transient three-phase induction motor model [30] is introduced here. Before applying this model for simulation, the following assumptions usually need to be made:

1. The constant temperature of the motor;
2. The three-phase winding is symmetrical, and the magnetic potential is distributed sinusoidal along the air gap;
3. Unsaturation of the magnetic circuit;
4. Without consideration of the hysteresis effect, the diaphragm effect, and the eddy current effect.

**Figure 1:**
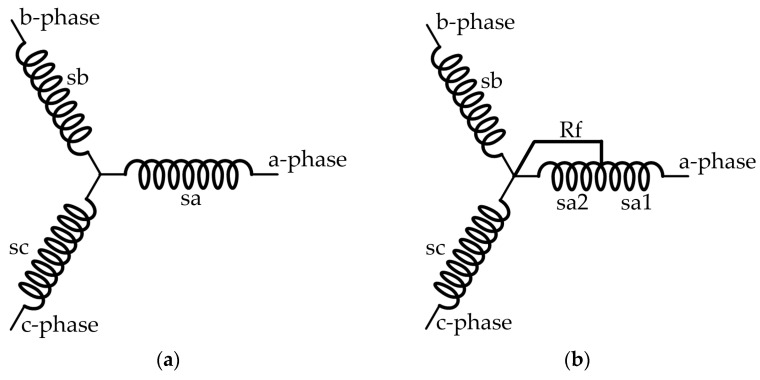
Stator winding diagram, (**a**) healthy state, (**b**) a phase ITSC fault.

When the motor is in a healthy state, the stator winding is symmetrically distributed in a 120-degree three-phase manner. When an ITSC fault occurs in the motor stator, the structure of the motor is no longer symmetrical. As shown in Figure 1, assuming that the three-phase stator winding of the motor is connected in Y configuration, an ITSC fault occurs in the a-phase winding, and the short circuit fault coefficient is $\mu ={sa}_{2}/({sa}_{1}+{sa}_{2})$, where ${sa}_{2}$ is the number of short-circuit turns and ${sa}_{1}+{sa}_{2}$ is the total number of turns of the a-phase winding. 

The voltage equation for the stator and rotor is

(1) $$\left[\begin{array}{c} u_{s}\\ u_{r} \end{array}\right]=\left[\begin{array}{cc} R_{s} & \\ & R_{r} \end{array}\right]\left[\begin{array}{c} i_{s}\\ i_{r} \end{array}\right]+p\left[\begin{array}{c} \psi_{s}\\ \psi_{r} \end{array}\right]$$

where $u_{s}={\left[u_{sa1}\;u_{sa2}\;u_{sb}\;u_{sc}\right]}^{T}$, $u_{sa1}$, $u_{sa2}$, $u_{sb}$, $u_{sc}$ are the instantaneous values of stator voltages for the normal winding part of phase a, the ITSC winding part of phase a, and the stator voltages for phases b and c, respectively. $u_{r}={\left[\;u_{ra}\;u_{rb}\;u_{rc}\right]}^{T}$**,** $u_{ra}$, $u_{rb}$, $u_{rc}$ are the instantaneous values of the rotor voltages of phase a, b, and c, respectively, and $u_{ra}=u_{rb}=u_{rc}=0$. $R_{s}={R_{s}diag[\left(1-\mu \right)\;\mu \;1\;1]}^{T}$, $R_{s}$ is the resistance value of each phase winding of the stator. $R_{r}=R_{r}I_{3\times 3}$,$\;R_{r}$ is the resistance value of each phase winding of the rotor. $i_{s}={\left[\;i_{sa}\;\left(i_{sa}-i_{f}\right)\;i_{sb}i_{sc}\right]}^{T},$ $i_{sa}$, $i_{sb}$, $i_{sc}$, $i_{f}$ are the instantaneous values of stator currents for phase a, b, c and short current, respectively. $i_{r}={\left[\;i_{ra}\;i_{rb}\;i_{rc}\right]}^{T}$, $i_{ra}$, $i_{rb}$, $i_{rc}$ are the instantaneous values of rotor currents in phases a, b, and c, respectively. p is the differential operator. $\psi_{s}={\left[\psi_{sa1}\;\psi_{sa2}\;\psi_{sb}\;\psi_{sc}\right]}^{T}$, $\psi_{sa1},\;{\;\psi }_{sa2},\;\psi_{sb},\;\psi_{sc}$ are the instantaneous values of the stator flux linkage for the normal winding part of phase a, the ITSC winding part of phase a, and the stator flux linkage for phases b and c, respectively. $\psi_{r}={\left[\psi_{ra}\;\psi_{rb}\;\psi_{rc}\right]}^{T}$,$\;{\;\psi }_{ra},\;\psi_{rb},\;\psi_{rc}$ are the instantaneous values of the rotor flux linkage for phases *a*, *b*, and *c*.

The magnetic linkage equations of the stator and rotor are

(2) $$\left[\begin{array}{c} \psi_{s}\\ \psi_{r} \end{array}\right]=\left[\begin{array}{cc} L_{ss} & L_{sr}\\ L_{rs} & L_{rr} \end{array}\right]\left[\begin{array}{c} i_{s}\\ i_{r} \end{array}\right]+\left[\begin{array}{c} i_{s}\\ i_{r} \end{array}\right]$$

where

(3) $$L_{ss}=L_{ls}diag{\left[\left(1-\mu \right)\;\mu \;0\;0\right]}^{T}+L_{ms}\left[\begin{array}{cccc} {\left(1-\mu \right)}^{2} & \mu (1-\mu ) & -\frac{1-\mu }{2} & -\frac{1-\mu }{2}\\ \mu (1-\mu ) & \mu^{2} & -\frac{\mu }{2} & -\frac{\mu }{2}\\ -\frac{\left(1-\mu \right)}{2} & -\frac{\mu }{2} & 1 & -\frac{1}{2}\\ -\frac{\left(1-\mu \right)}{2} & -\frac{\mu }{2} & -\frac{1}{2} & 1 \end{array}\right]$$

(4) $$L_{rr}=\left[\begin{array}{ccc} L_{lr}+L_{ms} & -\frac{L_{ms}}{2} & -\frac{L_{ms}}{2}\\ -\frac{L_{ms}}{2} & L_{lr}+L_{ms} & -\frac{L_{ms}}{2}\\ -\frac{L_{ms}}{2} & -\frac{L_{ms}}{2} & {L_{lr}+L}_{ms} \end{array}\right]$$

(5) $$L_{sr}={L_{rs}}^{T}=L_{ms}\left[\begin{array}{ccc} (1-\mu )cos\theta_{r} & \left(1-\mu \right)cos{(\theta }_{r}+\frac{2\pi }{3}) & \left(1-\mu \right)\cos(\theta_{r}-\frac{2\pi }{3})\\ \mu cos\theta_{r} & \mu cos{(\theta }_{r}+\frac{2\pi }{3}) & \mu cos(\theta_{r}-\frac{2\pi }{3})\\ cos(\theta_{r}-\frac{2\pi }{3}) & cos\theta_{r} & cos(\theta_{r}+\frac{2\pi }{3})\\ cos(\theta_{r}+\frac{2\pi }{3}) & cos{(\theta }_{r}-\frac{2\pi }{3}) & cos\theta_{r} \end{array}\right]$$

The stator mutual inductance is equal to the rotor mutual inductance, i.e., $L_{m}=L_{ms}=L_{mr}$. $\theta_{r}$ is the displacement angle between the motor’s corresponding stator phase and rotor phase.

The electromagnetic torque equation is

(6) $$T_{e}=-n_{p}L_{m}[\left(i_{sa}i_{ra}+i_{sb}i_{rb}+i_{sc}i_{rc}\right)sin\theta_{r}+\left(i_{sa}i_{rb}+i_{sb}i_{rc}+i_{sc}i_{ra}\right)sin(\theta_{r}+\frac{2\pi }{3})+\left(i_{sa}i_{rc}+i_{sb}i_{ra}+i_{sc}i_{rb}\right)sin(\theta_{r}-\frac{2\pi }{3})]$$

where $n_{p}$ is the number of magnetic pole pairs of the motor.

The torque balance equation is

(7) $$T_{e}=T_{L}+\frac{J}{n_{p}}\frac{d\omega_{r}}{dt}$$

where $T_{L}$ is the load torque, J is the moment of inertia, and $\omega_{r}$ is the angular velocity of the rotor.

## 3. Proposed Algorithm

**Figure 2:**
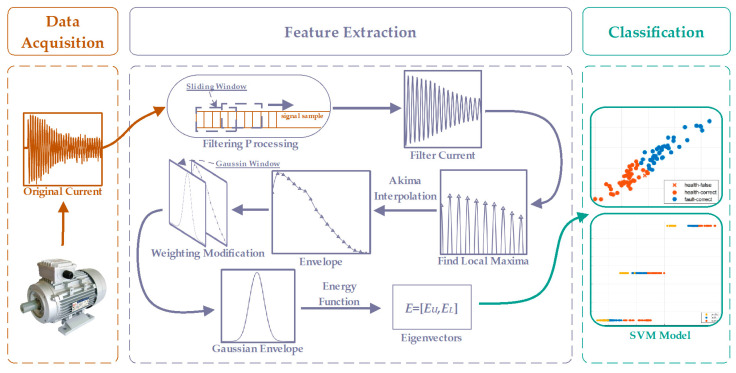
Proposed algorithm flow chart.

The flowchart of the proposed algorithm, as shown in Figure 2, consists of three steps: data acquisition, feature extraction, and classification. During the data acquisition process, the current signal of the induction motor was measured using a current sensor. In the feature extraction step, the raw current signal is first filtered using a sliding-average filter. Next, the upper and lower extreme points of the signal are calculated, and the upper and lower envelopes are obtained using the Akima interpolation function. Then, the Gaussian window is used to weigh the envelope and obtain the Gaussian envelope. Finally, the energies of the upper and lower Gaussian envelope are calculated using the energy formula to obtain the eigenvectors. In the classification step, the SVM model is used to classify the extracted eigenvectors. In the classification step, the SVM model is used to classify the extracted feature vectors of different health conditions (each color in the figure represents a health condition, such as health, early stage fault, and severe fault).

### 3.1. Sliding-Average Filter

After the motor current signal collection is completed, a sliding-average filter is used to smooth the original signal to reduce the impact of noise on the results. The calculation formula is as follows:

(8) $$f(i)=\frac{y\left(i-\frac{N}{2}\right)+y\left(i-\frac{N}{2}+1\right)+\ldots +y\left(i\right)+\ldots +y\left(i+\frac{N}{2}-1\right)+y(i+\frac{N}{2})}{N}$$

where *f(i)* represents the filtered value of the *i*-th point, *y(i)* represents the original value of the *i*-th point, and *N* represents the window width of the filter.

### 3.2. Envelope Extraction

The envelope is a commonly used method in signal analysis, widely employed in Empirical Mode Decomposition (EMD) [31] and Envelope Spectral Analysis (ESA) [32,33,34,35]. The core idea of the envelope is to reduce the dimension of the high-frequency signal and reflect the primary change trend of the signal. Common methods for envelope extraction include the Hilbert transform, the wavelet transform, and the interpolation-based techniques. Due to the complex and diverse frequency components of the original signal, traditional methods such as the Hilbert transform and wavelet transform can often struggle to achieve the desired results for envelope extraction. Interpolation, a classic mathematical method, can be used to extract the envelope with the following specific steps:

1. Calculate all the original signal’s maximum and minimum coordinate points.
2. Use the maximum and minimum coordinate points to obtain the upper and lower envelope through the interpolation function.

Compared with the Hilbert transform and wavelet transform, the envelope extracted by interpolation accurately passes through the extreme points of the original signal. Common methods of interpolation include Hermite interpolation, Lagrange interpolation, Newton interpolation, and Akima interpolation, among others. Akima interpolation [36] has unique advantages. Like the cubic spline interpolation method, the Akima interpolation method takes into account the effects of the derivative values of the elements. However, cubic spline interpolation is sensitive to outliers in the input data, even a small outlier can have a significant impact on the interpolation result. In contrast, Akima interpolation is relatively robust when dealing with outliers and is less likely to be disturbed, showing excellent robustness. Furthermore, cubic spline interpolation can cause oscillations, where the derivative of the interpolation function changes rapidly at the connection points, resulting in discontinuities or non-smoothness in the interpolation result. On the other hand, Akima interpolation can generate a continuously smooth interpolation function, avoiding this oscillation issue. Finally, Akima interpolation uses local linear functions, which makes it computationally faster, and especially suitable for interpolating large-scale datasets.

The Akima interpolation method is a method of interpolation between two measured points. In addition to using these two measured values, it also requires the observed values on the four adjacent measured points of these two points. In fact, interpolating between two measured points requires a total of six measured points. The specific calculation process is as follows:

1. The original data are ($x_{i}$, $y_{i}$)(i=1,2,3…,n), assuming that the curve y=f(x) meets $y_{i}=f(x_{i})$. Meanwhile, any two adjacent data points are approximated using a cubic polynomial.
2. Curve y needs to meet four conditions:

(9) $$\left\{\begin{array}{c} y_{i}=f(x_{i})\\ y_{i+1}=f(x_{i+1})\\ y_{i}^{\prime }=k_{i}\\ y_{i+1}^{\prime }=k_{i+1} \end{array}\right.$$

where $k_{i}$ is the slope of the point *i*. Therefore, the unique cubic polynomial can be determined, and the whole curve obtained is also smooth.

3. The expression for the cubic polynomial is as follows:

(10) $$y=p_{0}+p_{1}\left(x-x_{i}\right)+{p_{2}(x-x_{i})}^{2}+{p_{3}(x-x_{i})}^{3}$$

where

(11) $$\left\{\begin{array}{c} p_{0}=y_{i}\\ p_{1}=k_{i}\\ p_{2}=\frac{3(y_{i+1}-y_{i})/(x_{i+1}-x_{i})-2k_{i}-k_{i+1}}{\left(x_{i+1}-x_{i}\right)}\\ p_{3}=\frac{k_{i}+k_{i+1}-2(y_{i+1}-y_{i})/(x_{i+1}-x_{i})}{{\left(x_{i+1}-x_{i}\right)}^{2}} \end{array}\right.$$

$k_{i}$ is determined by the measured values at points i−2, i−1, i+1, i+2, and the calculation formula is

(12) $$\left\{\begin{array}{c} k_{i}=(\left|m_{i+1}-m_{i}\right|m_{i-1}+\left|m_{i-1}-m_{i-2}\right|m_{i})/(\left|m_{i+1}-m_{i}\right|+\left|m_{i-1}-m_{i-2}\right|)\\ m_{i}=(y_{i+1}-y_{i})/(x_{i+1}-x_{i}) \end{array}\right.$$

The equation does not hold when $\left|m_{i+1}-m_{i}\right|+\left|m_{i-1}-m_{i-2}\right|=0$. Therefore, when this situation occurs, Akima states $t_{i}$ = ($m_{i+1}+m_{i}$) or $t_{i}=m_{i}$.

When dealing with endpoint problems, adding two prediction points outside the endpoint is necessary. Based on the four conditions that y needs to meet, it is not essential to specifically determine the positions of these two points, but rather to calculate their slope k.

### 3.3. Gaussian Window Weighting and Calculation of Envelope Energy

**Figure 3:**
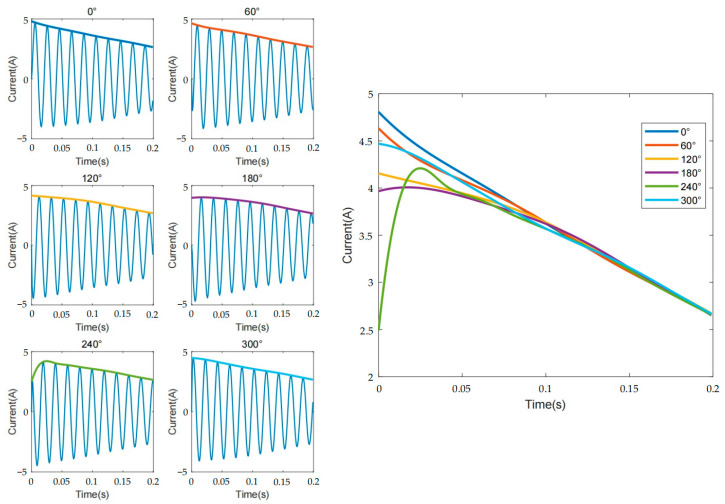
The start-up current and envelope with different initial phase angles.

Due to variations in the timing of the induction motor connected to the power source, the initial phase angle of the three-phase voltage source may differ. As a result, the initial phase angle of the collected current signal may also vary. In this scenario, obtaining the corresponding envelope using Akima interpolation results in significant differences at the starting point, also known as end effects, as shown in Figure 3.

Therefore, it is necessary to allocate weights to the envelope data accordingly to minimize the impact of end effects and amplify the amplitude variation of the starting current. In this paper, the entire envelope undergoes weighted modification using the Gaussian window function. The coefficients of the Gaussian window are computed from the following equation:

(13) $$w\left(n\right)=e^{-\frac{1}{2}{\left[\alpha \frac{n}{(L-1)/2}\right]}^{2}}$$

where L represents the window length, and α represents the width factor, and its exact correspondence with the standard deviation of a Gaussian probability density function is σ=(L−1)/(2α).

After processing with the Gaussian window weighting, the upper and lower Gaussian envelope data are obtained as $U=\left\{\left(x_{1},y_{1}\right),\left(x_{1},y_{1}\right),\left(x_{1},y_{1}\right)\ldots \left(x_{1},y_{1}\right)\right\}$, respectively.

The amplitude analysis of the signal includes both amplitude and energy aspects. Signal energy refers to the total energy of a signal within a certain period of time and is commonly used in signal processing, communication systems, image processing, and other fields. It can be used to measure a signal’s strength, quality, and other aspects. The mathematical definition of energy is

(14) $$E_{j}=\sum_{i=1}^{n}{\left|y_{i}\right|}^{2}$$

where y=U or L. $y_{i}$ represents the amplitude of the first data point in the upper or lower filtering envelope.

Finally, the eigenvectors $E=[E_{U},E_{L}]$ will be used for fault detection.

### 3.4. Support Vector Machine

SVM is a machine learning classification method based on the theory of structural risk minimization proposed by Vapnik and his colleagues [37]. It is applicable to pattern classification and handling nonlinear regression problems. SVM has the advantage of achieving excellent performance in the small sample and binary classification problems. Its fundamental idea is to define a classification hyperplane as the decision surface, which can accurately distinguish the training data’s positive and negative sample points while maximizing the geometric margin between the hyperplane and the samples. The derivation process is as follows:

1. Assuming the training dataset is $D=\{\left(X_{1},Y_{1}\right),\left(X_{2},Y_{2}\right),\ldots (X_{n},Y_{n})\}$ and linearly separable, where $X_{i}\in R^{d}$, $X_{i}$ are eigenvectors of dimension d, and $Y_{i}\in \{-1,+1\}$ is the class of the sample, when $Y_{i}=-1$, it is a negative example, and when $Y_{i}=+1$, it is a positive example.
2. Assuming the classification hyperplane is y=x+b, in order to maximize the geometric margin between the data points and the classification hyperplane, the Lagrange method is introduced to solve the optimization problem, which can be expressed as

(15) $$\left\{\begin{array}{c} min\frac{1}{2}\sum_{i=1}^{n}\sum_{j=1}^{n}y_{i}y_{j}\alpha_{i}\alpha_{j}\left(x_{i}\cdot x_{j}\right)-\sum_{j=1}^{n}\alpha_{j}\\ s.t.\;\;\sum_{i=1}^{n}y_{i}\alpha_{i}=0,\alpha_{i}\geq 0,i=1,2,\ldots n \end{array}\right.$$

3. After obtaining the optimal solution $\alpha^{*}=(\alpha_{1}^{*},\alpha_{2}^{*},\ldots ,\alpha_{n}^{*})$, and selecting a positive component $\alpha_{j}^{*}$ of $\alpha^{*}$, the parameters of the hyperplane can be calculated as follows:

(16) $$b^{*}=y_{i}-\sum_{i=1}^{n}y_{i}\alpha_{j}^{*}(x_{i}\cdot x_{j})$$

4. The decision function can be constructed as

(17) $$f\left(x\right)=sgn(\sum_{i=1}^{n}y_{i}\alpha_{j}^{*}x_{i}x+b^{*})$$

where sgn() is the sign function which returns +1 for positive values and −1 for negative values.

## 4. Mathematical Simulation

Mathematical simulations were performed on the start-up current signals of the induction motor with varying degrees of ITSC faults to evaluate the proposed method. Table 1 shows the basic parameters of the induction motor simulation model.

Taking the ITSC in the A-phase winding of an electric motor as an example, simulations were performed for a total of 54 scenarios. These scenarios included three different fault levels for $\mu_{1}=0$, $\mu_{2}=0.01$, $\mu_{3}=0.03$ (representing healthy, early-stage ITSC fault and severe fault condition, respectively), six different initial phase angles for the $\varphi_{1}=0{}^{\circ}$, $\varphi_{2}=60{}^{\circ}$, $\varphi_{3}=120{}^{\circ}$, $\varphi_{4}=180{}^{\circ}$, $\varphi_{5}=240{}^{\circ}$, $\varphi_{6}=300{}^{\circ}$, and three different load conditions at torque values of 0, 0.25, and 0.5 Nm.

**Figure 4:**
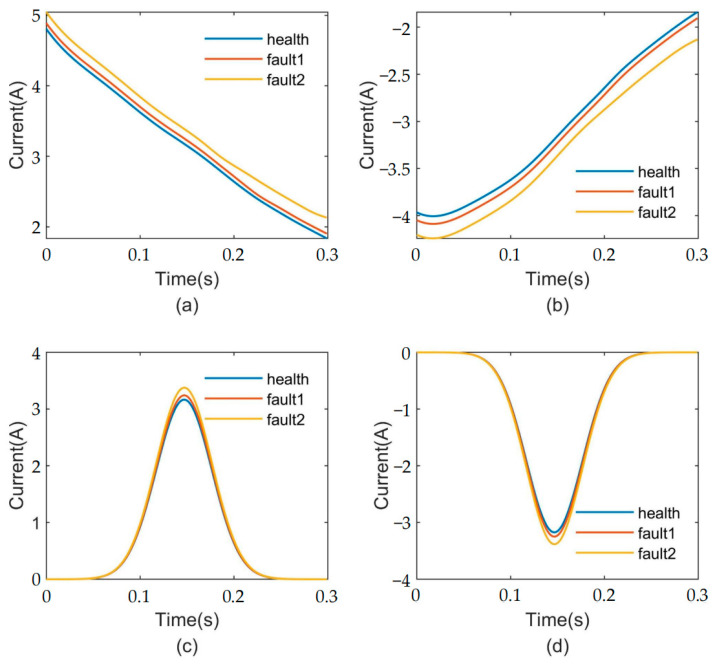
The feature extraction process for different fault levels: (**a**) Upper envelope. (**b**) Lower envelope. (**c**) Gaussian upper envelope. (**d**) Gaussian lower envelope.

The upper and lower envelopes were obtained by using the Akima interpolation function. Next, the upper and lower envelopes are separately weighted and processed with a Gaussian window of α=5 to obtain the upper and lower Gaussian envelopes. Figure 4 shows the feature extraction process for different fault levels.

The energy of the upper and lower Gaussian envelopes was calculated for each situation, and corresponding health status labels were assigned to each sample as feature vector samples. Specifically, the feature vector samples with μ = 0 were labeled as ‘health’, those with μ = 0.01 were labeled as ‘fault1′, and those with μ = 0.03 were labeled as ‘fault2′. Table 2 presents the Gaussian envelope energies for each situation.

**Figure 5:**
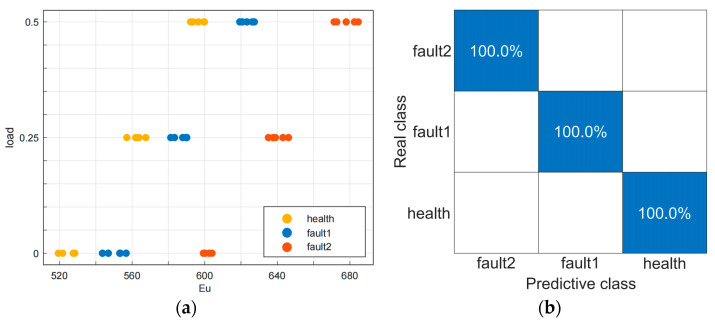
(**a**) Scatter plot. (**b**) Confusion matrix.

An amount of 95% of the eigenvector samples were randomly selected as input to train the model, while the remaining 5% of the eigenvector samples were used as a testing dataset to validate the trained model. The training and validation accuracy of the model both reached 100%. The proposed approach not only enables the detection of ITSC faults in induction motors but also identifies the severity of the faults. Figure 5a,b show the scatter plot and confusion matrix of the trained model, respectively.

## 5. Results and Discussion

### 5.1. Experiment Setup

**Figure 6:**
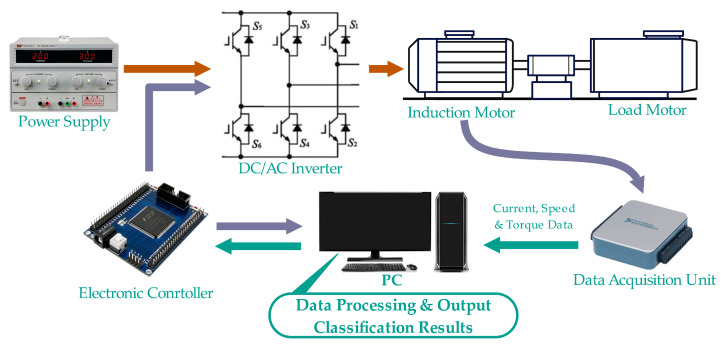
Basic structure of the experiment.

To verify the effectiveness of the proposed method, an electric motor performance testing platform was established for motor current data acquisition experiments on start-up. The basic structure of the experiment is shown in Figure 6.

**Figure 7:**
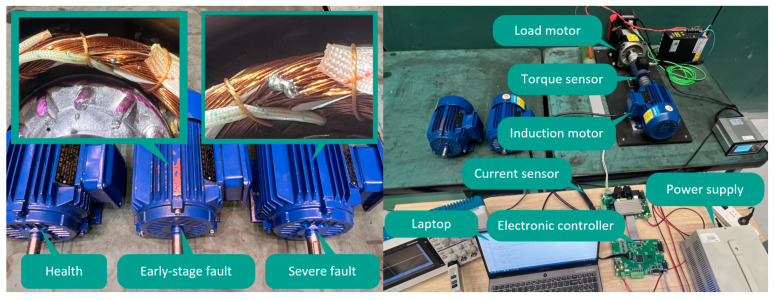
Experimental induction motor and laboratory setup.

In the experiment, three induction motors of the exact specifications were used as test objects, including a healthy motor, an early-stage fault motor, and a severe fault motor. The early-stage fault motor is made by manually scraping off the insulation layer of the adjacent coils of the U phase and then using welding technology to weld the two coils together, resulting in early ITSC faults. The severe fault motor was made by welding the six adjacent coils in the u-phase using the same method. The basic parameters of this motor are a rated power of 1 hp, two pairs of poles, and a rated speed of 1400 rpm. The equipment of the entire experimental platform includes a load motor and its corresponding controller capable of providing 7.5 Nm, a torque sensor with a range of 15 Nm and an accuracy of 0.2% Fs, a TCP0030A current sensor (TEKTRONIX) with a bandwidth of 120 MHz, a maximum effective current of 30A and sensitivity of 1 mA, a TBS2000B oscilloscope (TEKTRONIX), a laptop computer equipped with MATLAB, a voltage source, an experimental induction motor, and its corresponding controller. The sampling frequency of the current sensor was set to 20 kHz, and the motor was connected to an AC voltage with a frequency of 20 Hz through the controller. The experimental induction motor and laboratory setup are shown in Figure 7.

### 5.2. Results Analysis

**Figure 8:**
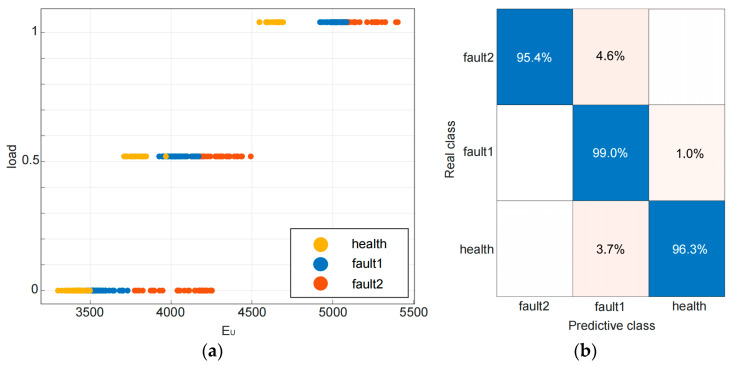
(**a**) Scatter plot of experimental training model. (**b**) Confusion matrix of experimental training model.

The experimental platform has collected a data set of u-phase start-up current signals for healthy, early-stage fault and severe fault motors, which includes three scenarios under no load, 0.52 Nm, and 1.04 Nm loads, totaling 450(50×3×3) sets of data. To ensure the effectiveness of the model, a randomized approach was employed to divide the dataset into two distinct subsets: an 80% sample used for training and a residual 20% sample reserved solely for testing. This approach was chosen to reduce potential problems of under-fitting or over-fitting that might impede the model’s predictive performance. In addition, a data set of current signals for the steady-state operation of induction motors was obtained under three different load conditions. Using the proposed method, the upper and lower envelope energies of each group of data in the training set are extracted as feature vectors, and corresponding state labels are set for the samples to obtain feature samples $S_{i}=[E_{Ui},E_{Li},load,type]$, where $S_{i}$ represents the *i*-th sample, $E_{Ui}$ and $E_{Li}$ represent the upper and lower Gaussian envelope energy of the *i*-th sample, load = 0, 0.52 or 1.04 represents the load situation of the induction motor, and type = health, fault1 or fault2 represents the status label of the sample (‘fault1′ refers to early-stage fault, while ‘fault2′ indicates severe fault). The feature dataset was inputted into the SVM model for training, and finally, the model was verified using the test feature dataset. The trained model achieved a detection accuracy of 96.3%, 95.4%, and 99% for health, early-stage fault, and severe fault, respectively, with an average accuracy of 96.9% and a validation average accuracy of 95.3%. Figure 8a,b, respectively, show the scatter plot and confusion matrix plot of the trained model.

**Figure 9:**
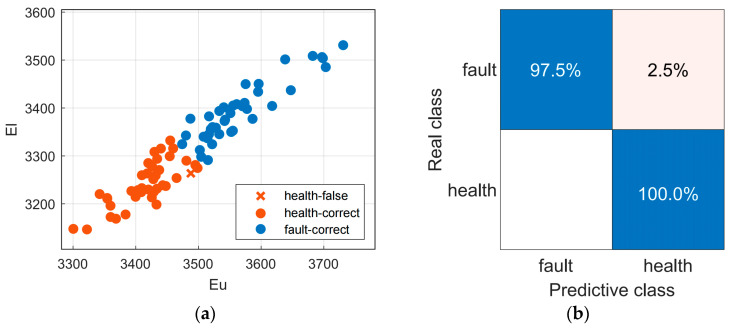
(**a**) Scatter plot. (**b**) Confusion matrix.

In addition, we conducted further experimental analysis on early fault detection under no-load conditions. The experiment will use the start-up current dataset of health and early-stage fault under no-load conditions. An amount of 80% of the sample data are randomly selected as the training set, and the remaining 20% are used as the validation set, which is input into the classification model for training and validation. Figure 9a,b, respectively, show the scatter plot and confusion matrix of the training model. The proposed method achieved 100% and 97.5% accuracy in detecting health and faults in the training model, with an average accuracy of 98.8%. In validation, it reached an average accuracy of 100%.

### 5.3. Discussion

#### 5.3.1. Classification Performance Analysis

To verify the early-stage fault detection superiority of the proposed method under no-load conditions, the other classification machine learning methods, including Logistic Regression (LR), k-Nearest Neighbor (KNN), and Neural Network (NN), will be compared. Table 3 shows the comparison results. The results show that the average accuracy of using LR, KNN, and NN to train classification models was 96.2%, 97.5%, and 98.8%, respectively. The average accuracy of LR and KNN is lower than SVM, while NN has the same accuracy as SVM but a longer training time. From the above, it can be seen that the proposed method has excellent performance in SVM classification and achieved satisfactory results in this dataset. In addition, it is worth noting that although NN requires a relatively long training time, its accuracy after training is also excellent, and it has great potential on the basis of the proposed method.

#### 5.3.2. Limitations of the Proposed Method

Although the fault detection and determination of the severity of ITSC in induction motors has been achieved by utilizing the envelope energy of the starting current in this paper, several limitations to our study that should be acknowledged.

1. The instability of the voltage source will have a specific impact on the motor’s start-up current, resulting in a decrease in the fault detection accuracy of the proposed method. Therefore, in future research, the stability characteristics of the voltage need to be considered.
2. When ITSC faults occur in other phases, they will affect the starting current of the measured phase, resulting in misjudgment of the location of the short circuit fault. This means that measuring single-phase current cannot achieve the position determination of ITSC faults.

## 6. Conclusions

In this study, to solve the problem of difficult detection of early-stage ITSC faults in the three-phase induction motor, we propose an ITSC fault detection method based on the envelope energy of the start-up current. The results demonstrate that the proposed method exhibits excellent performance in the detection of ITSC faults and the determination of the severity in induction motors. The detection accuracy of the proposed method training model for health, early-stage fault, and severe fault reached 96.3%, 95.4%, and 99%, respectively, with an average accuracy of 96.9% and a validation accuracy of 95.3%. Furthermore, to verify the early-stage fault detection superiority of the proposed method under no-load conditions, the other classification machine learning methods were compared. The average accuracy of the SVM training model for the proposed method is 98.8%, while the average accuracies of the LR, KNN, and NN training models are 96.2%, 97.5%, and 98.8%, respectively.

Our future work will focus on determining the location of the fault. We may consider adding current sensors to other phases to improve the performance of the proposed method. Then, we will use voltage signals to reduce the impact of voltage instability on the proposed method. In addition, we will consider incorporating temperature sensors to monitor the motor and analyze the influence of temperature factors on the proposed method. Finally, we will study more effective machine learning classification models to improve the accuracy of fault detection.

## Figures and Tables

**Table 1:** Basic parameters of the induction motor simulation model.

Parameters	Values
Frequency f(Hz)	50
Rated voltage U(V)	220
Number of poles $n_{p}$	2
Stator resistance $R_{s}(\Omega )$	20.63
Rotor resistance $R_{r}(\Omega )$	20.69
Stator leakage inductance $L_{ls}(H)$	0.0151
Rotor leakage inductance $L_{lr}(H)$	0.0141
Mutual inductance between stator and rotor $L_{m}(H)$	0.347
Inertia $J(kg\cdot m^{2})$	0.0066

**Table 2:** The Gaussian envelope energies for each situation.

Type		Load					
		No Load		0.25 Nm		0.5 Nm	
		Upper Energy	Lower Energy	Upper Energy	Lower Energy	Upper Energy	Lower Energy
Health	0°	527.553	530.990	563.514	567.522	599.831	604.131
	60°	519.365	521.687	555.176	557.106	592.212	593.567
	120°	528.407	527.650	563.809	561.980	599.486	596.512
	180°	530.990	527.553	567.522	563.514	604.131	599.831
	240°	521.687	519.365	557.106	555.176	593.567	592.212
	300°	527.650	528.407	561.980	563.809	596.512	599.486
Fault 1	0°	553.208	556.694	590.093	594.171	627.316	631.706
	60°	543.654	546.890	581.301	583.363	619.326	620.762
	120°	553.397	552.692	589.689	587.894	626.246	623.280
	180°	556.694	553.208	594.171	590.093	631.706	627.316
	240°	546.890	543.654	583.363	581.301	620.762	619.326
	300°	552.692	553.397	587.894	589.689	623.280	626.246
Fault 2	0°	600.357	604.015	638.967	643.109	677.946	682.491
	60°	599.295	602.505	635.106	637.718	671.325	672.771
	120°	607.993	607.472	646.167	644.522	684.601	681.725
	180°	604.015	600.357	643.109	638.967	682.491	677.946
	240°	602.505	599.295	637.718	635.106	672.771	671.325
	300°	607.472	607.993	644.522	646.167	681.725	684.601

**Table 3:** Comparison results.

Method	Accuracy (Validation)	Training Time
Proposed Features + SVM	**98.8%**	**0.5395 s**
Proposed Features + LR	96.2%	0.8597 s
Proposed Features + KNN	97.5%	0.6569 s
Proposed Features + NN	**98.8%**	1.2627 s

## Data Availability

Data available on request from the authors.
